# Hierarchical Macro-Mesoporous Silica Monolithic Tablets as a Novel Dose–Structure-Dependent Delivery System for the Release of Confined Dexketoprofen

**DOI:** 10.1021/acs.molpharmaceut.2c00834

**Published:** 2022-12-19

**Authors:** Marta Kozakiewicz-Latała, Dominik Marciniak, Karolina Krajewska, Adrianna Złocińska, Krystian Prusik, Bożena Karolewicz, Karol P. Nartowski, Wojciech Pudło

**Affiliations:** † Department of Drug Forms Technology, Faculty of Pharmacy, Wroclaw Medical University, Borowska 211, Wroclaw50-556, Poland; ‡ Institute of Materials Engineering, University of Silesia in Katowice, 75 Pulku Piechoty 1A, Chorzow40-007, Poland; § Silesian Center for Education and Interdisciplinary Research, University of Silesia in Katowice, 75 Pulku Piechoty 1A, Chorzow40-007, Poland; ∥ Department of Chemical Engineering and Process Design, Silesian University of Technology, Gliwice44-100, Poland; ⊥ Laboratory of Elemental Analysis Structural Research, Wroclaw Medical University, Borowska 211, Wroclaw50-556, Poland

**Keywords:** macropore-favored crystallization, hierarchical porous drug carriers, silica monolithic tablets, controlled and adjustable release of dexketoprofen, dexketoprofen crystallization in macroporous structures

## Abstract

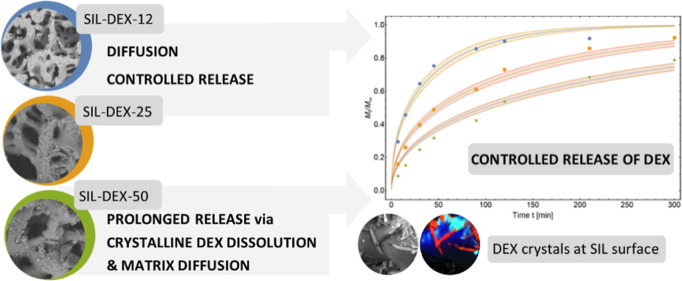

This study reports the application of hierarchical porous monoliths as carriers for controlled and dose-adjustable release of model pharmaceutical (dexketoprofen, DEX). The synthesis and detailed characterization of the hierarchical porous scaffolds are provided before and after the adsorption of three doses of DEX—a widely used nonsteroidal anti-inflammatory drug. The drug incorporated in the mesopores of silica was stabilized in an amorphous state, while the presence of macropores provided sufficient space for drug crystallization as we demonstrated via a combination of powder X-ray diffraction, differential scanning calorimetry, and imaging techniques (scanning electron microscopy and EDX analysis). Drug release from silica matrices was tested, and a mechanistic model of this release based on the Fick diffusion equation was proposed. The hierarchical structure of the carrier, due to the presence of micrometric macropores and nanometric mesopores, turned out to be critical for the control of the drug phase and drug release from the monoliths. It was found that at low drug content, the presence of an amorphous component in the pores promoted the rapid release of the drug, while at higher drug contents, the presence of macropores favored the crystallization of DEX, which naturally slowed down its release. Both the hierarchical porous structure and the control of the drug phase (amorphous and/or crystalline) were proven important for adjustable (fast or prolonged) release kinetics, desirable for effective pharmacotherapy and patient compliance. Therefore, the developed materials may serve as a versatile formulation platform for the smart manipulation of drug release kinetics.

## 1 Introduction

For several years, materials with a hierarchical porous structure have attracted great interest in the scientific community due to their similarities to structures found in nature.^1−3^ Such materials have large channel-like macropores (1–50 μm) and smaller mesopores (2–50 nm), guaranteeing high surface area.^4,5^ These properties make the materials suitable for the application as enzyme carriers,^6^ precursors of chemical microreactors,^7−10^ or monolithic columns for chromatography.^11,12^ Little space is devoted to the use of these materials as pharmaceutical carriers, especially for the controlled release of poorly water-soluble drugs that account for nearly 50% of currently marketed drugs and an estimated 70% of pipeline active pharmaceutical ingredients (APIs).^13^

Insufficient solubility, limited stability in body fluids, or limited cellular uptake of some pharmaceutical compounds necessitate the search for novel drug carriers. The first attempts for the application of porous (and nonporous) silica materials as drug carriers were reported in 1972 by Lach and Monkhouse.^14^ The materials were proven promising for improving the dissolution of drugs that are poorly soluble in water.^15^ Since that discovery, the use of different types of ordered mesoporous silicas as drug carriers has subsequently been investigated.^16−20^ The possibility of using hierarchical mesoporous silica (HMS) as matrices for drug delivery systems with adjustable release capacity could significantly improve the therapeutic efficacy of poorly soluble APIs.^21^ Controlled or modified release of medicinal substances seems to be the optimal dosage method, increasing the effectiveness of pharmacotherapy and patient adherence. A particular form of controlled release is adjustable drug release,^22^ in which the profile is adapted to the therapeutic needs of the patient.^23^ The hierarchical porous structure, due to high flexibility in the independent regulation of the size and shape of macro- and mesopores, seems to naturally fit in as a precursor of carriers for adjustable drug delivery. The internal pore architecture of the matrix is critical for controlled drug release. The presence of different types of pores in the silica matrix results in different properties of the carriers (pore volume, surface area, and pore arrangement), enabling the facilitation of the ability to control the release of the loaded drugs.^24^ Although several studies have demonstrated the effect of porosity on the state of confined pharmaceuticals, the drug-release process from porous matrices is yet to be fully understood. The encapsulation of pharmaceuticals inside nanoporous materials provides a novel approach for the manipulation of the drug state from amorphous to nanocrystalline.^25,26^ Smart manipulation of the state of a drug (e.g., amorphous or crystalline) may provide a way to increase the dissolution rate and, as a consequence, the bioavailability of the drug. The control over a drug phase can be achieved via its incorporation inside the pores of silica scaffolds. For example, the stabilization in the amorphous state of fast crystallizing compounds (flufenamic acid and tolbutamide) via nanoscale confinement within mesoporous silica carriers has been demonstrated by our group.^25,26^ In both cases, the control over a phase of confined molecules was achieved via control over the pore size and/or the content of organic species inside the pores, resulting in fully amorphous or hybrid amorphous/nanocrystalline composites.^26−28^

The extended release of doxorubicin hydrochloride from HMSs was presented by Wu et al.^24^ The difference in the pore parameters between the outer and inner structure of the produced particles was proven critical for the controlled release of the API from the materials. Wang et al. reported HMS microspheres as carriers for the controlled release of indomethacin.^29^ The produced HMS with the incorporated drug had organized mesochannels and large intraparticulate mesopores. A rapid release of indomethacin was observed from high-mesoporosity microspheres, while a prolonged release was observed from low-mesoporosity microspheres. Zůza et al.^21^ used melt loading for the incorporation of ibuprofen into silica particles with three levels of porosity. The mesoporous structure enabled the stabilization of the amorphous drug due to the spatial confinement effect, while the presence of macropores enabled the rapid flow of the molten drug through the particles. The possibility of incorporation of seven APIs of different physicochemical properties into silica carriers by solvent evaporation was demonstrated by Šoltys et al.^30^ The APIs incorporated in the mesoporous materials remained in an amorphous state, while loading into hierarchical meso-/macroporous silicas resulted in loading level-dependent crystallization of the compounds.^30^ Recently, our group presented novel hierarchical porous monoliths, which thanks to the high accessibility and sorption capacity, allowed us to control the release of APIs adsorbed in its pores.^31^ Careful control of the synthetic conditions allows tailoring the size of both macro- and mesopores, enabling the adjustment of the properties of the carrier to drug molecules or specific release profiles. The formulation of the materials at the stage of synthesis allows designing the shape of the monoliths without the use of excipients, facilitating and reducing the cost of formulation and possibly increasing the safety of pharmacotherapy by reducing side effects caused by excipients.^32^

This study reports the application of hierarchical porous monoliths as carriers for controlled and dose-adjustable release of dexketoprofen (DEX)—a widely used nonsteroidal anti-inflammatory drug (NSAID). The synthesis and detailed characterization of the hierarchical porous scaffolds is provided before and after the adsorption of three doses of DEX. The phase of the drug incorporated in the mesopores of silica was assessed via a combination of powder X-ray diffraction (PXRD), differential scanning calorimetry (DSC), and imaging techniques [scanning electron microscopy (SEM) and EDX analysis]. Drug release from silica matrices was tested, and a mechanistic model of this release based on Fick’s diffusion equation was proposed. Both the hierarchical porous structure and the control of the drug phase (amorphous and/or crystalline) were proven important for adjustable (fast or prolonged) release kinetics, desirable for effective pharmacotherapy and patient compliance. Therefore, the developed materials may serve as a versatile formulation platform for the smart manipulation of drug release kinetics.

## 2 Materials and Methods

### 2.1 Materials

(S)-(+)-Ketoprofen (DEX) was supplied by Pol-Aura (Olsztyn, Poland), and ethanol 96% (v/v) was supplied by Honeywell (Brazil). Tetraethoxysilane (TEOS), poly(ethylene glycol) (PEG) with an average molecular weight of 35000 g/mol, and cetyltrimethylammonium bromide (CTAB) were provided by Sigma-Aldrich (Poland). Nitric acid (65%) and ammonia solution (33%) were supplied by Avantor Performance Materials Poland S.A. (Gliwice, Poland). All other agents were provided by Sigma-Aldrich (Poland). Chemicals were used without further purification.

### 2.2 Methods

#### 2.2.1 Carrier Synthesis

Materials were prepared according to the procedure given by Smått et al.^5^ and modified by Pudlo et al.^33^ In a typical synthesis of silica monolithic tablets, PEG was dissolved in 1 M nitric acid, and then TEOS was added dropwise. When the solution was homogeneous in the whole volume, CTAB was dissolved. The molar ratios of components TEOS/HNO_3_/H_2_O/PEG/CTAB were as follows 1:0.25:14.7:0.54:0.029. The sol generated was sonicated for 5 min, then gelled, and aged for 7 days at 40 °C. After that time, gels were treated with 1 M ammonia for 12 h at 90 °C, then neutralized using 0.1 M nitric acid and water, and, finally, dried for 5 days at 60 °C and calcined at 550 °C at a heating rate of 1 °C/min. Before pharmaceutical testing, silica monoliths were divided into tablets with a height of 3 mm and a diameter of 5 mm.

#### 2.2.2 Drug Loading and Drug Content Determination

The nine samples of silica monolithic tablets were soaked for 2 h in sealed vials in ethanolic solutions of DEX at the concentrations of (A) 56.25 mg/mL, (B) 112.5 mg/mL, and (C) 225.0 mg/mL. The samples were then dried to constant weight using the Moisture Analyzer MAX 60 (Radwag, Radom, Poland) for approximately 50 min. The content of the loaded drug in the silica monoliths was determined independently using high-performance liquid chromatography (HPLC) and thermogravimetric analysis [see Materials and Methods (2.2.5.) for details]. For HPLC analysis, the drug-loaded monolith was placed in a vial containing 5 mL of ethanol and left for 24 h under constant stirring to extract the drug. From the resulting solutions, 20 μL of the sample was withdrawn, diluted with 1 mL of water, and analyzed by HPLC according to the protocol described in Section 2.2.9.

#### 2.2.3 Pore Structure Characterization

Materials were analyzed by low-temperature nitrogen adsorption and scanning (SEM) and transmission (TEM) electron microscopy to determine their porous structure. SEM (TM 3000 Hitachi) was used to examine the macroporous structure. The EDX chemical mapping and point spectra measurements were performed using a JEOL SSD detector attached to a JSM 7100F high-resolution field emission gun scanning electron microscope. TEM (S/TEM Titan 80-300) was used to examine the mesoporous structure. The specific surface area and pore size distribution were determined from low nitrogen adsorption (ASAP Micromeritics 2020). The Brunauer–Emmett–Teller (BET) specific surface area (*S*_BET_) and the volume of monolayer coverage were determined using the BET equation.^34^ The pore volume *versus* diameter distribution was calculated by analyzing the desorption branch of the isotherm using the Barrett–Joyner–Halenda method.^35^

#### 2.2.4 DSC

DSC 214 Polyma (Netzsch, Selb, Germany) equipped with an IntraCooler was used to perform DSC analysis. The samples (5–7 mg) were weighed on aluminum pans with pierced lids. The analysis was recorded at a heating rate of 5 °C/min and a nitrogen flow rate of 50 mL/min over a temperature range from −50 to 200 °C. Each sample was tested in a triple cycle (heating, cooling, and heating). The content of crystalline DEX in the formulations was determined using the heat of fusion of DEX as detected in different SIL-DEX formulations.

#### 2.2.5 Thermogravimetric Analysis

TG 209 F1 Libra (NETZSCH) with an automatic sample feeder was used to perform thermogravimetric analysis (TGA). The samples (10–15 mg) were weighed into Al_2_O_3_ crucibles and analyzed over a temperature range from 25 to 800 °C with a temperature increment of 5 °C/min. The test was conducted under a nitrogen atmosphere with the gas flow rate fixed at 50 mL/min.

#### 2.2.6 Powder X-ray Diffraction

A D2 Phaser diffractometer (Bruker AXS) with a one-dimensional LYNXEYE strip detector and Cu Kα radiation (1.5418 Å) was used to determine the presence and phase of crystalline DEX in the monoliths. A step size of 0.02° 2θ and an irradiation time of 1 s/step were used in the analysis using the Bragg–Brentano (θ/2θ) horizontal geometry between 5 and 36° 2θ. A divergence slit of 0.2 mm, an antiair-scatter screen of 1 mm, and a Ni filter were used during measurement.

#### 2.2.7 FTIR Spectroscopy

The Nicolet iS50 spectrometer (Thermo Scientific, Waltham, MA, USA) with an attenuated total reflectance was used for Fourier-transform infrared spectroscopy (FTIR) analysis of the obtained materials. Spectra were recorded at wavelengths from 400 to 4000 cm^–1^ with 32 scans per sample and a 4 cm^–1^ resolution.

#### 2.2.8 Drug Dissolution Study

The dissolution studies were performed using a USP type II apparatus Hanson SR-8 Plus (Hanson, Chatsworth, CA, USA). Samples were placed in 1000 mL vessels containing dissolution media (phosphate buffer pH = 6.8) at 37 ± 2 °C and stirred at a rate of 50 rpm. At specified time intervals (7.30, 15.00, 22.30, 30.00, 45.00, 60.00, 90.00, 120.00, 150.00, 180.00, 210.00, and 240.00 min), the apparatus was used to withdraw 3 mL samples through the in line 0.45 μm filters (Quality Lab Accessories LLC, Telford, PA, USA) while replenishing with 3 mL of the pure medium.

#### 2.2.9 High-Performance Liquid Chromatography

Ultra-HPLC (UHPLC, Thermo Scientific UltiMate 3000, Dionex Corporation, Sunnyvale, CA, USA) and an Ascentis Express RP-18, 10 cm × 4.6 mm, 2.7 μm (Supelco) column was used for drug content determination. A UHPLC assay was carried out using gradient elution with a flow rate of 0.8 mL/min, a column temperature of 30 °C, and a mobile phase of water 0.1% FA (A) and acetonitrile 0.1% FA (B) with the detector set at the wavelength of 256 nm. 10 μL of the sample was injected on the column.

#### 2.2.10 Drug Release Modeling

Due to the fact that the release of the active substance from the tested formulations is controlled by the rate of its diffusion in the porous structure of the matrix, a mathematical description of this process was analyzed using a mechanistic physical model based on an accurate, analytical solution of the Fick diffusion equation assuming a finite, cylindrical geometry of the system (eq 1)^36−39^
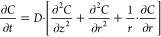
1where *z* is half the height of the cylinder, *r* is the radius of its base, and *D* is the diffusion coefficient.

A detailed mathematical description of the modeling is presented in the Supporting Information (Section 2. Drug Release Modelling). Additionally, the dissolution efficiency [DE (see the Supporting Information, Section 2. Drug Release Modelling), a statistical parameter characterizing the release profile independent of the adopted model] was determined for each of the release profiles, enabling comparison of the drug release kinetics from three silica matrices: SIL-DEX-12, SIL-DEX-25, and SIL-DEX-50. The mean values of the DE determined for the three tested systems were compared with each other using the parametric ANOVA with posthoc Fisher’s NIR test of least significant difference. The normality of the distributions of the compared variables was assessed by the Shapiro–Wilk test and the homogeneity of their variance by the Levene test. All the compared variables met the assumptions of normal distribution and homogeneity of variance. The results of the parametric analysis of variance are presented in Table 3 and SI Figure S10.

## 3 Results and Discussion

### 3.1 Drug Loading and Textural Properties of Hierarchical Silica Materials

**Figure 1:**
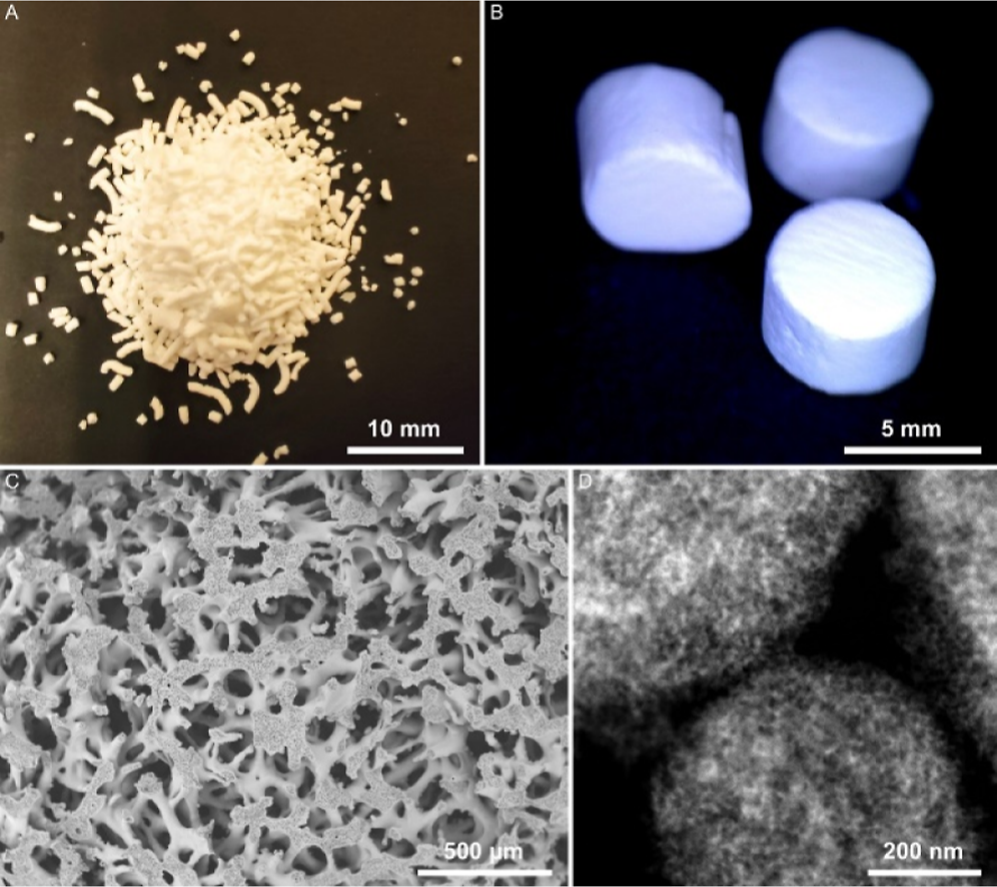
Silica monolithic pellets (A) and tablets (B). Structure of silica tablets with open-through macropores (C) and mesopores (D) [SEM (C) and TEM (D) micrographs].

**Figure 2:**
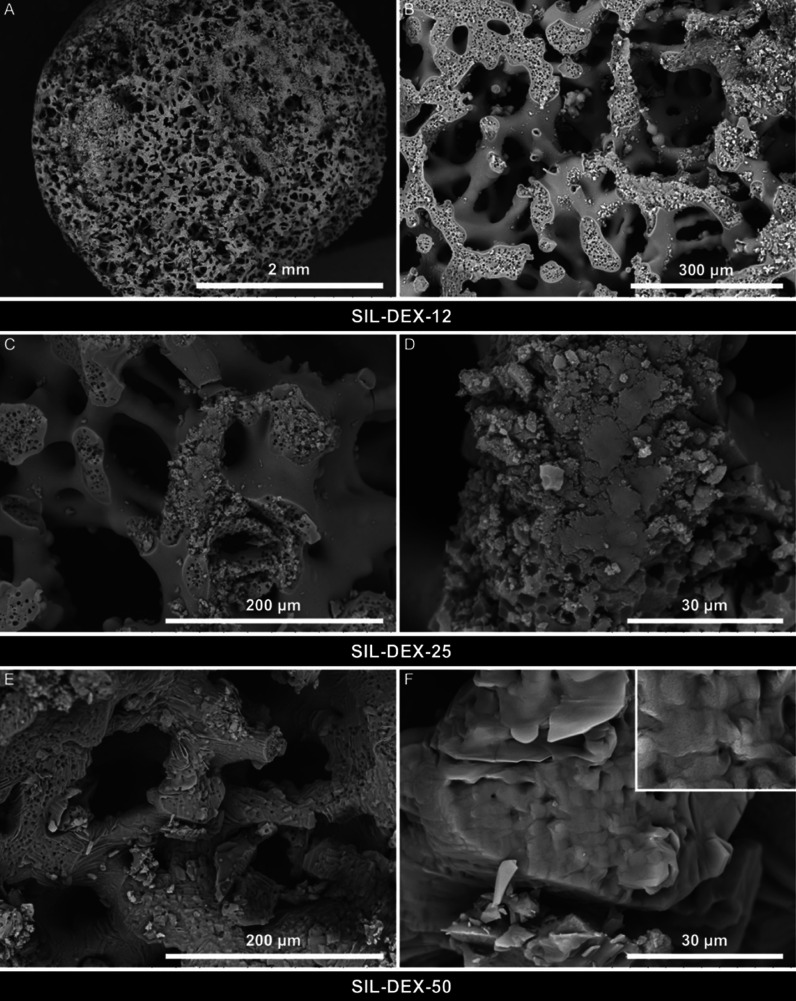
SEM micrographs of the macroporous structure of silica monolithic tablets with incorporated (s) (+)-ketoprofen: SIL-DEX-12 (A,B), SIL-DEX-25(C,D), and SIL-DEX-50 (E,F).

**Figure 3:**
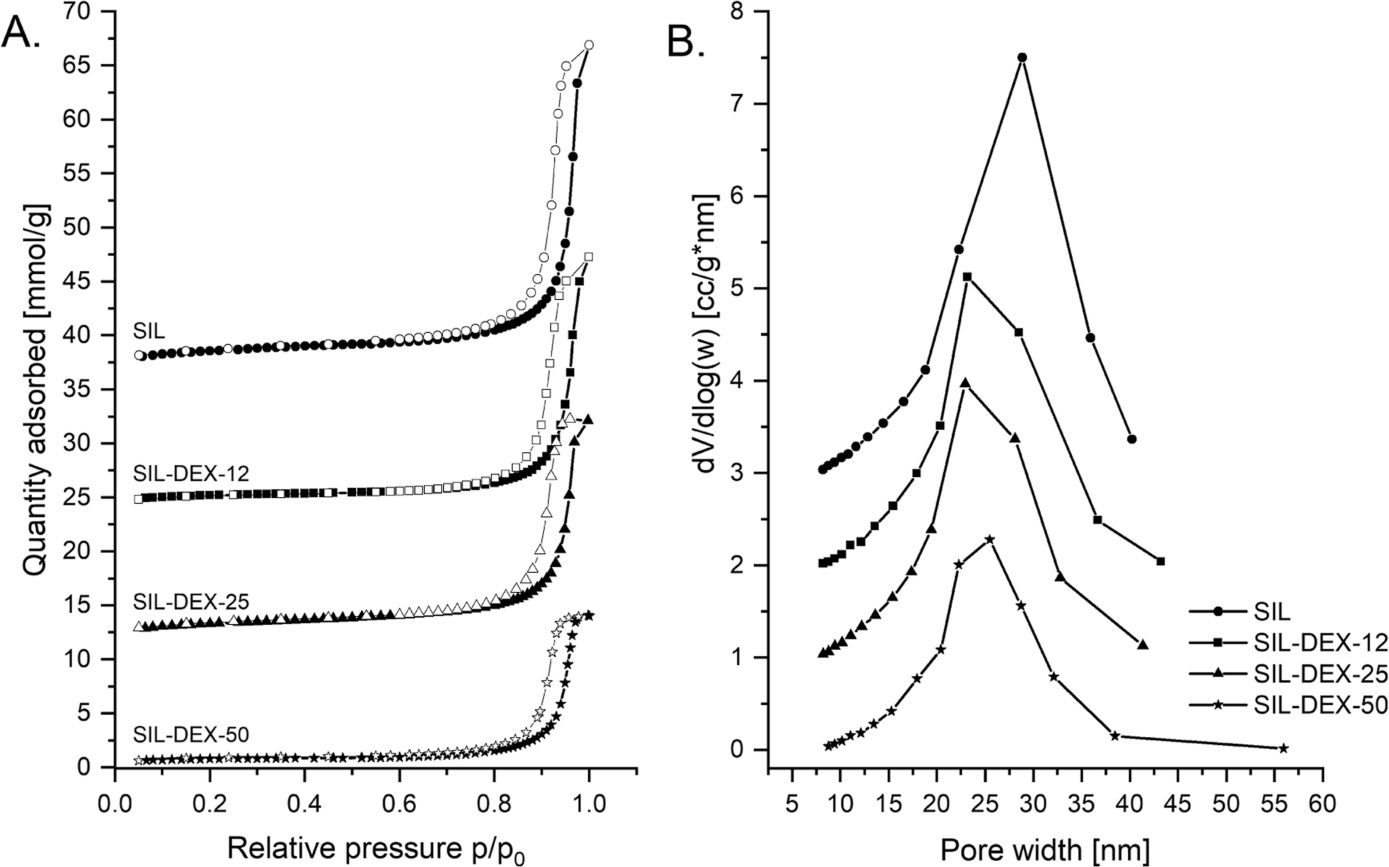
Adsorption isotherms (A) and BJH pore size distributions (B) for silica and silica tablets (SIL) loaded with (s)-(+)-ketoprofen (SIL-DEX-12, SIL-DEX-25, and SIL-DEX-50). The isotherms are offset by 12 mmol/g for clarity. The adsorption points are labeled with filled points, while the desorption points are marked with open shapes.

We synthesized silica monolithic tablets with the hierarchical macro-mesoporous structure (Figure 1) and impregnated them with various doses of (s)-(+)-ketoprofen (DEX), obtaining SIL-DEX-12, SIL-DEX-25, and SIL-DEX-50 formulations (Table 1). The presence of macropores was visualized by SEM (Figures 1C and 2), while the presence of mesopores was confirmed by TEM (Figure 1D) and low-temperature nitrogen adsorption (Figure 3). The obtained SIL materials had a mesopores volume of 0.95 cm^3^/g and a surface area of 187 m^2^/g. The mesopores’ diameter was determined at ca. 29 nm using the BJH method. Upon drug incorporation, both the surface area as well as mesopores’ volume displayed a gradual decrease, indicating that the drug was incorporated into the mesopores (Table 1). These changes were corroborated by a slight decrease in the pore size diameter (Figure 3).

**Table 1:** Drug Content and Textural Properties of Silica Monolithic Tablets Loaded with Different Doses of DEX

formulation	SIL-DEX composite [mg] ± SD	DEX [mg] ± SD	DEX [wt %]	BET surface area [m^2^/g]	volume of mesopores [cm^3^/g]	content of crystalline DEX in the composite (wt %)
SIL	14.0 ± 0.1			187	0.95	0
SIL-DEX-12	16.3 ± 0.6	2.6 ± 0.2	∼16	105	0.73	2.48
SIL-DEX-25	18.2 ± 0.4	5.2 ± 0.2	∼28	87	0.69	8.66
SIL-DEX-50	23.3 ± 0.2	14.0 ± 2.1	∼60	53	0.49	9.15

**Figure 4:**
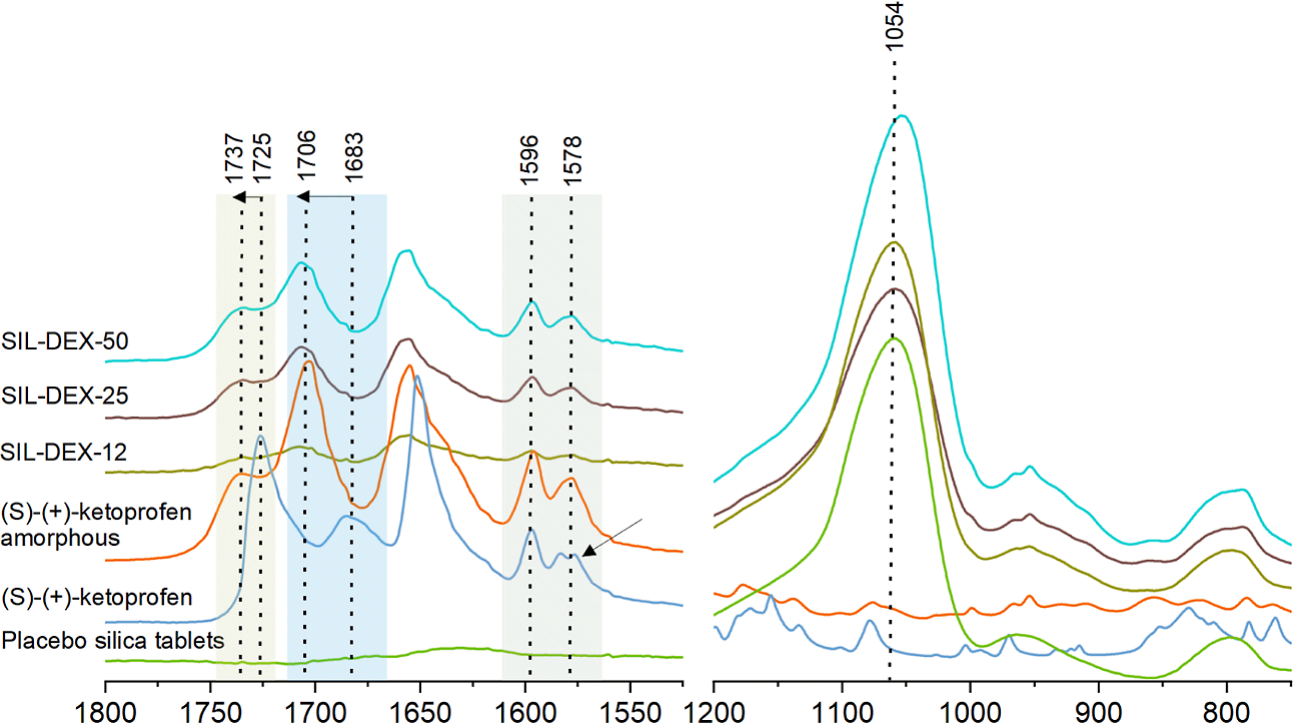
FTIR spectra of (s)-(+)-ketoprofen, silica, and silica monoliths with three doses of (s)-(+)-ketoprofen.

**Figure 5:**
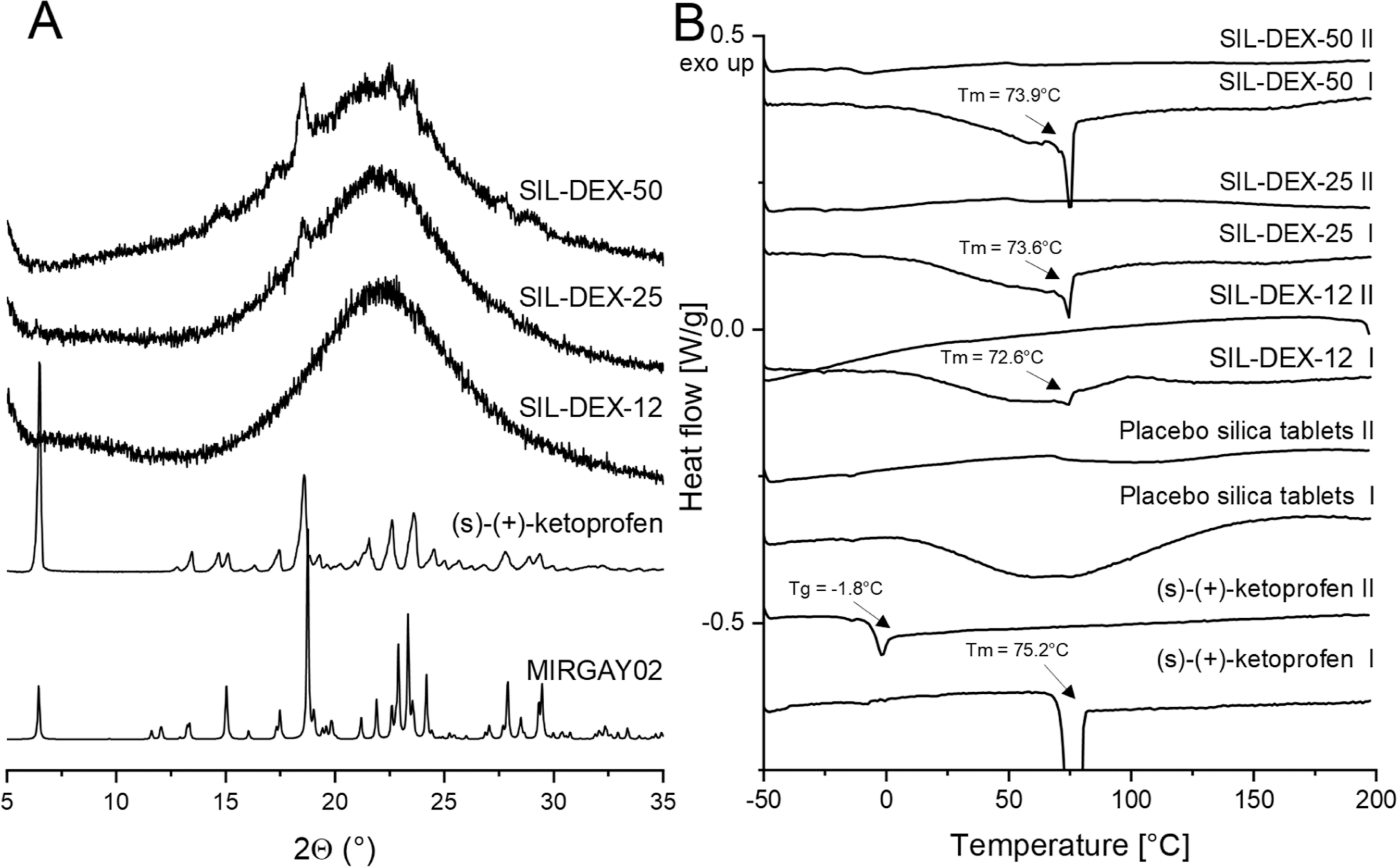
(A) XRD pattern and (B) DSC thermogram of silica monolithic tablets with incorporated (s)-(+)-ketoprofen. The figure is zoomed in to display the DEX melting peak in silica composites. The figure with a full scale that includes the melting peak of DEX can be found in the Supporting Information (Figure S6). The calculated PXRD pattern of (s)-(+)-ketoprofen (CSD ref. code MIRGAY02) is given for comparison. The first heating and second heating in the DSC thermograms are labeled as (I) and (II), respectively.

The presence of the drug in the monolithic tablets was also confirmed by programmed thermogravimetry (TG; see the Supporting Information, Figure S1), FTIR spectroscopy (Figure 4 and Figure S3), powder X-ray analysis (PXRD, Figure 5A), and DSC (Figure 5B). The drug content in the monoliths was in the range from 2.6 ± 0.2 to 14.0 ± 2.1 mg for SIL-DEX-12 and SIL-DEX-50, respectively, as determined using HPLC analysis (Figure S2 and Tables 1 and S1).

### 3.2 Drug Phase and its Crystallization at the Silica Pores

In the FTIR spectrum of crystalline (s)-(+)-ketoprofen in the spectral range from 1525 to 1800 cm^–1^ the peak centered at 1725 cm^–1^ corresponds to the C=O stretching mode of the carboxylic acid group of DEX molecules stabilized in the crystalline state in the form of infinite hydrogen-bonded chains (Figure 4).^40^ The peak at 1649 cm^–1^ is assigned to the C=O stretching of the ketone group, while the peaks between 1625 and 1550 cm^–1^ are characteristic of the C=C stretching of the phenyl group^41^ that correspond to the peaks at 1596 and 1578 cm^–1^ marked in Figure 4. Once incorporated in the silica pores, the IR spectra of DEX underwent substantial changes matching the spectrum of amorphous DEX. The IR bands of amorphous and silica-confined DEX are broader as compared to the peaks of the crystalline drug. This is due to the lack of long-range ordering characteristics of amorphous solids. The peak at 1725 cm^–1^, corresponding to linear OH···O interactions in the crystalline DEX, underwent a shift to a lower wave number (1706 cm^–1^) due to the change in the molecular environment. This could be attributed to the formation of cyclic dimmers in an amorphous state based on the density functional theory (DFT), supported by the assignment of the ketoprofen IR spectrum.^41^ Champeau et al. distinguished ketoprofen molecules as monomers and cyclic and linear dimers based on the distinct peaks in the spectrum of the drug dissolved in supercritical CO_2_. The spectral features observed in this work match the description of DFT-calculated spectral features reported by Champeau et al.^41^

Although FTIR spectra showed that the drug is present predominately in its amorphous state inside the pores of silica monoliths, PXRD and DSC (Figures 4 and 5) proved that the drug partially crystallizes on the surface of the silica pores. This was further confirmed by comparative analysis of images of drug structures deposited on the pore walls (Figure 2E,F) using the EDX SEM technique (Figures S4 and S5).

The presence of a crystalline form of (s)-(+)-ketoprofen was confirmed in two prepared formulations (SIL-DEX-25 and SIL-DEX-50), as indicated by broad, low-intensity peaks observed in the baseline of PXRD diffractograms (angular values 2θ = 19, 22.5, and 23.5°, Figure 5A). The detected peaks match the pattern of starting (s)-(+)-ketoprofen, indicating no change in the crystalline structure of the drug upon loading into the pores of silica monoliths. In addition, DSC thermograms (Figure 5B) confirmed the presence of the crystalline drug in all formulations including SIL-DEX-12. The melting point (mp) of (s)-(+)-ketoprofen was detected at 73.9 °C (SIL-DEX-50), 73.6 °C (SIL-DEX-25) and 72.6 °C (SIL-DEX-12). The mp of DEX loaded in silica monoliths was slightly lower as compared to the mp of neat DEX at 75.2 °C. This can be related to the confinement effect^42^ along drug crystal/silica surface interactions, as seen in the SEM images (Figure 2D and Figures S4 and S5). The content of crystalline DEX in all three materials was determined from the heat of fusion of the DEX melting peak. The crystalline content varied from 2.48% in SIL-DEX-12 composites to 9.15% in SIL-DEX-50 composites. This is also corroborated by changes in glass transition temperature detected in the DSC heat–cool–heat cycle. The *T*_g_ of neat DEX equals −1.8 °C, while the SIL-DEX-50 composite displays a *T*_g_ value of −8.1 °C (Figure 5B). The decrease of the glass transition temperature of confined solids was extensively discussed by McKenna et al.^43−45^ and is a well-established phenomenon in mesoporous systems.

**Scheme 1:**
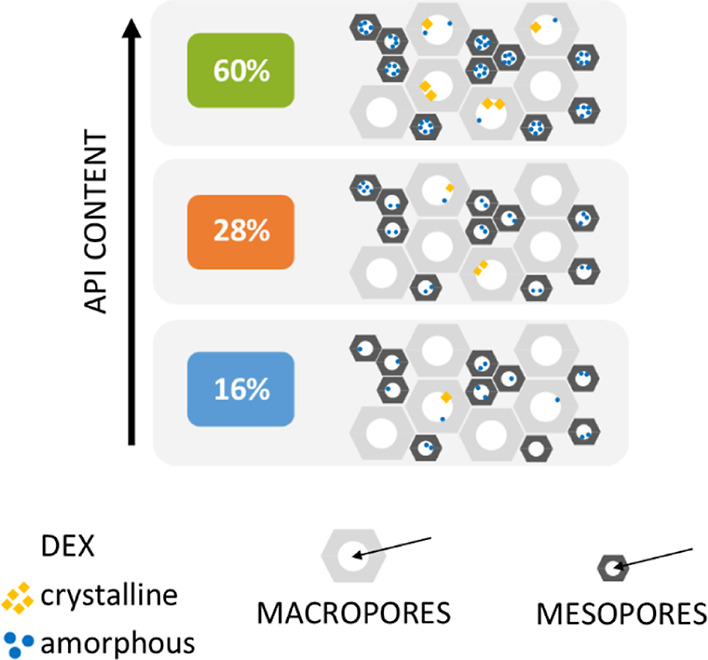
Drug Content-Dependent Distribution and Crystallization of DEX within Meso- and Macropores

The presence of crystalline components within the materials was confirmed in the SEM images (Figure 2C–F) which show a change in the morphology of the macropore walls for SIL-DEX-25 and SIL-DEX-50 samples caused by the deposition of drug crystals on their surface. Additionally, the crystallization of the drug on the surface of the SIL-DEX-50 material was confirmed by the EDX/SEM technique by mapping the entire surface of the sample (Figure S4) or by collecting data from a selected point of the surface (Figure S5). The structure of the macropores, especially the porous surface consisting of chains of condensing silica, favors the phenomenon of spatially limited crystallization, as reported for several pharmaceuticals.^26,28,46−48^ A similar situation, although to a much lesser extent, is applied to the SIL-DEX-12 composite. In this case, a small part of the macropore surface changes due to the lower content of (s)-(+)-ketoprofen, which is deposited within the macropores of the material and forms crystalline domains locally at the pore surface (Scheme 1). Hence, due to the low degree of crystallization and the limited number of crystallization sites, the corresponding reflexes do not appear in the XRD patterns (Figure 5).

### 3.3 Drug Content Controls the Diffusion from the Silica Monoliths

**Figure 6:**
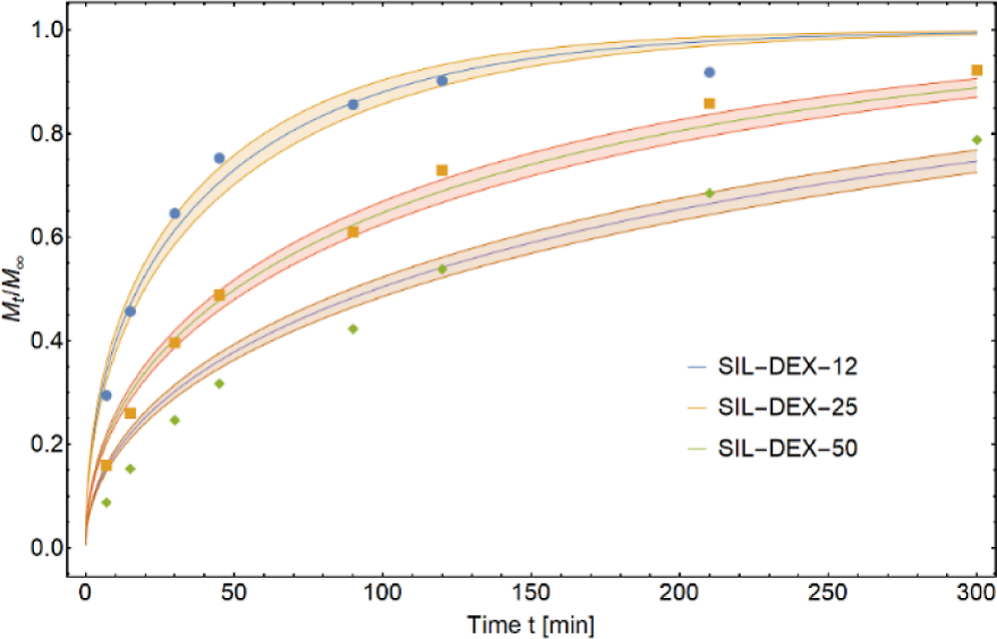
Comparison of the release kinetics (diffusion rate) of the API from the three matrices F1 (SIL-DEX-12), F2 (SIL-DEX-25), and F3 (SIL-DEX-50) with the average fractions released after time *t* and the functions of the best fit of the diffusion model (eq S5) to empirical data.

The drug release kinetics was modeled using the Fick diffusion equation (Figure 6, Table 2, and SI Section S2, and Table S2). The performed statistical analysis of the quality of fit of the used diffusion model to the obtained empirical data clearly indicates a very high degree of correlation of the analyzed release profiles with the estimated function. The calculated values of the correlation coefficients *R* in each case oscillated around the value of ≈0.99, which is satisfactory taking into account the estimated nonlinear function being an exact solution of the Fick diffusion equation that was characterized by only one parameter—the diffusion coefficient *D*. The determined estimators of the diffusion coefficients *D* in all three performed nonlinear iterations were highly statistically significant. The *t*-test estimated that the significance levels p for the estimators *D̂* were smaller than 1 × 10^–20^. In addition, the diffusion coefficient values, respectively, 1.3 × 10^–6^, 4.6 × 10^–7^, and 2.4 × 10^–7^ cm^2^/s, fully comply with the theoretical predictions according to which the diffusion of molecules of similar size in an unrestricted water environment should be characterized by diffusion coefficients D of the order 1 × 10^–5^ cm^2^/s.^49,50^ On the other hand, the diffusion rate in monolithic polymer matrices usually oscillates around the value of the order 1 × 10^–10^ cm^2^/s.^49−51^ All the values of the D diffusion coefficients calculated on the basis of the applied mechanical diffusion model, as predicted, are in the range from 1 × 10^–5^ to 1 × 10^–10^ cm^2^/s.

**Table 2:** Statistical Evaluation of the Nonlinear Estimation Performed Using the Least-Square Method Based on the Diffusion Model Used (See Equation S5). Part 1 (See Table S2 for Part 2)

formulation	estimator*D̅*[cm^2^/min]	diffusion coefficient *D*[cm^2^/s]	standard error SE	statistics *t*	significance level *p*	AIC	BIC	*R*^2^
SIL-DEX-12	0.0000795001	1.325 × 10^–6^	4.86 × 10^–6^	16.36	8.48 × 10^–21^	–111.8	–106.2	0.991
SIL-DEX-25	0.0000276809	4.613 × 10^–7^	1.34 × 10^–6^	20.65	5.28 × 10^–30^	–166.8	–160.3	0.991
SIL-DEX-50	0.0000142291	2.372 × 10^–7^	6.71 × 10^–7^	21.19	3.56 × 10^–32^	–170.8	–163.9	0.988

The structural properties of the obtained materials correspond well to the differences in the release of (s)-(+)-ketoprofen from macro-mesoporous silica carriers (Figure 6). The obtained values of the diffusion coefficients allow for a significant differentiation of the described release profiles and correlate with the other determined statistical parameters (DE release efficiency), which were used to compare the pharmaceutical availability of DEX incorporated in the three analyzed cylindrical matrices (Table 3). The comparison of the DE of DEX from three investigated materials displayed statistically significant differences between the release profiles. The highest dissolution efficiency (DE_SIL-DEX-12_ = 0.854 ± 0.059) was obtained for the SIL-DEX-12 composite, and it was about 13% higher than the dissolution efficiency of SIL-DEX-25 formulation (DE_SIL-DEX-25_ = 0.755 ± 0.079) and 42% higher than the DE of SIL-DEX-50 composite (DE_SIL-DEX-50_ = 0602 ± 0.057).

**Table 3:** Results of the Parametric ANOVA

material	dissolution efficiency (average) DE	*n*	standard deviation SD
SIL-DEX-12	0.854	6	0.059
SIL-DEX-25	0.755	6	0.0799
SIL-DEX-50	0.602	6	0.0579
Sum	0.737	18	0.124

Variable	SS effect	Df effect	MS effect	SS error	Df error	MS error	*F*-Statistics	*p*-value
DE	0,195	2	0,0972	0,0650	15	0,00434	22,44	3.1 × 10^–5^

We observe the fastest release for monolithic tablets with the lowest drug content (SIL-DEX-12) and the slowest with the highest drug content (SIL-DEX-50). In the presented model of diffusion, it is manifested by the decrease in the diffusion coefficient of DEX from 1.325 × 10^–6^ to 2.372 × 10^–7^ cm^2^/s (Table 2), that is, almost 10 times. As the drug loading within the pores increases (SIL-DEX-25 and SIL-DEX-50 DEX carriers), due to the increase in drug concentration in the impregnating solution, local supersaturation of the drug solution occurs, causing crystallization on the macropores’ surface (Figure 2), and the drug release is being extended. This can be a result of (i) limited access of the dissolution medium into the pores that are now filled with the deposited drug (as shown in nitrogen physisorption analysis) and (ii) the presence of crystalline components within the matrix that displays a slow dissolution rate. Control of the crystal size and material crystallinity is a well-known strategy, enabling slowing down of the drug dissolution and extending its release.^52^

The shape and size of the mesopores present in the structure of the monolithic tablet can also affect the release profile of the drug from obtained composites. The API, during the loading process, diffuses through macropores to mesopores, contributing to the reduction of their volume and average size from approx. 30 (silica) to 25 or 20 nm (for SIL-DEX materials) (Figure 3). This leads to a reduction in the mesopores’ volume of silica tablets and the related reduction of the BET specific surface area of carriers from 187 m^2^/g (neat silica carrier) to 105, 87, and 53 m^2^/g (SIL-DEX-12, SIL-DEX-25, and SIL-DEX-50, respectively) (Table 1). The adsorption results (Figure 3) prove good penetration of the drug through the macro-mesoporous network and preferential deposition of the drug in the mesopores upon carrier impregnation from ethanol solutions. Depositing the drug in mesopores allows for a significant extension of the process of drug release over time because its release requires the penetration of the solvent into the macro-mesoporous structure, its dissolution, diffusion of drug molecules from the mesopores to macropores, and finally, mass transport from the macropores to the dissolution medium.

## 4 Conclusions

This work demonstrated for the first time the dose–structure-dependent release of (s)-(+)-ketoprofen from silica monolithic tablets with a hierarchical porous structure. Using porous silica composites loaded with different contents of DEX (from ca. 16 to 60 wt %), we demonstrated that amount of the loaded drug in combination with the hierarchical porous structure allows controlling the rate of the drug diffusion through the macro-mesopore system. For all evaluated materials, less than 10 wt % of the crystalline drug was detected at the surface of macropores, indicating preferential deposition of DEX in the mesopores regardless of the drug concentration in a loading solution. The deposition of the drug in the mesopores along with its partial crystallization at the macropores enabled us to produce novel drug delivery materials with dose-controlled drug release. The presented approach enables us to better understand the drug transport phenomena in hierarchically porous monoliths and can have general applicability for the design of novel porous scaffolds for the delivery of drugs and other molecules of industrial importance.

## Supporting Information Available

DEX content in the materials determined using HPLC and TGA, HPLC calibration curve for drug content determination, FTIR spectra, SEM/EDX mapping of the materials’ surface, full-scale DSC thermogram, details on the DEX release modeling, and extensive statistical evaluation (PDF)
