# Supplementary material for: Hierarchical Macro-Mesoporous Silica Monolithic Tablets as a Novel Dose–Structure-Dependent Delivery System for the Release of Confined Dexketoprofen
Source: Mol Pharm. 2022 Dec 19;20(1):641–9. doi: 10.1021/acs.molpharmaceut.2c00834 (PMC9811460; doi:10.1021/acs.molpharmaceut.2c00834)
Supplement: Supplementary file 1 — mp2c00834_si_001.pdf [file mp2c00834_si_001.pdf]

SUPPORTING INFORMATION

Hierarchical macro-mesoporous silica monolithic tablets as novel dose-structure dependent delivery system for the release of confined dexketoprofen

Marta Kozakiewicz-Latała[1], Dominik Marciniak[1], Karolina Krajewska[1], Adrianna Złocińska[5], Krystian Prusik[2,3], Bożena Karolewicz[1], Karol P. Nartowski[1]*, Wojciech Pudło[4]*

[1]Department of Drug Forms Technology, Faculty of Pharmacy, Wroclaw Medical University, Borowska 211, Wroclaw, Poland

[2]Institute of Materials Engineering, University of Silesia in Katowice, 75 Pulku Piechoty 1A, Chorzow, Poland

[3]Silesian Center for Education and Interdisciplinary Research, University of Silesia in Katowice, 75 Pulku Piechoty 1A, Chorzow, Poland

[4]Department of Chemical Engineering and Process Design, Silesian University of Technology, Gliwice, Poland

[5]Laboratory of Elemental Analysis Structural Research, Wroclaw Medical University, Borowska 211, Wroclaw, Poland

*correspondence:

Wojciech Pudło: wojciech.pudlo@polsl.pl

Karol P. Nartowski: karol.nartowski@umw.edu.pl

SECTION 1.Material characterization.

Table S1. (S)(+) ketoprofen content in silica monolithic tablets.

[mg]	1	2	3	Average	SD
**50 DEX**	16.25	11.20	14.62	14.02	2.10
**25 DEX**	5.35	5.31	4.87	5.18	0.22
**12.5 DEX**	2.70	2.38	2.69	2.59	0.15

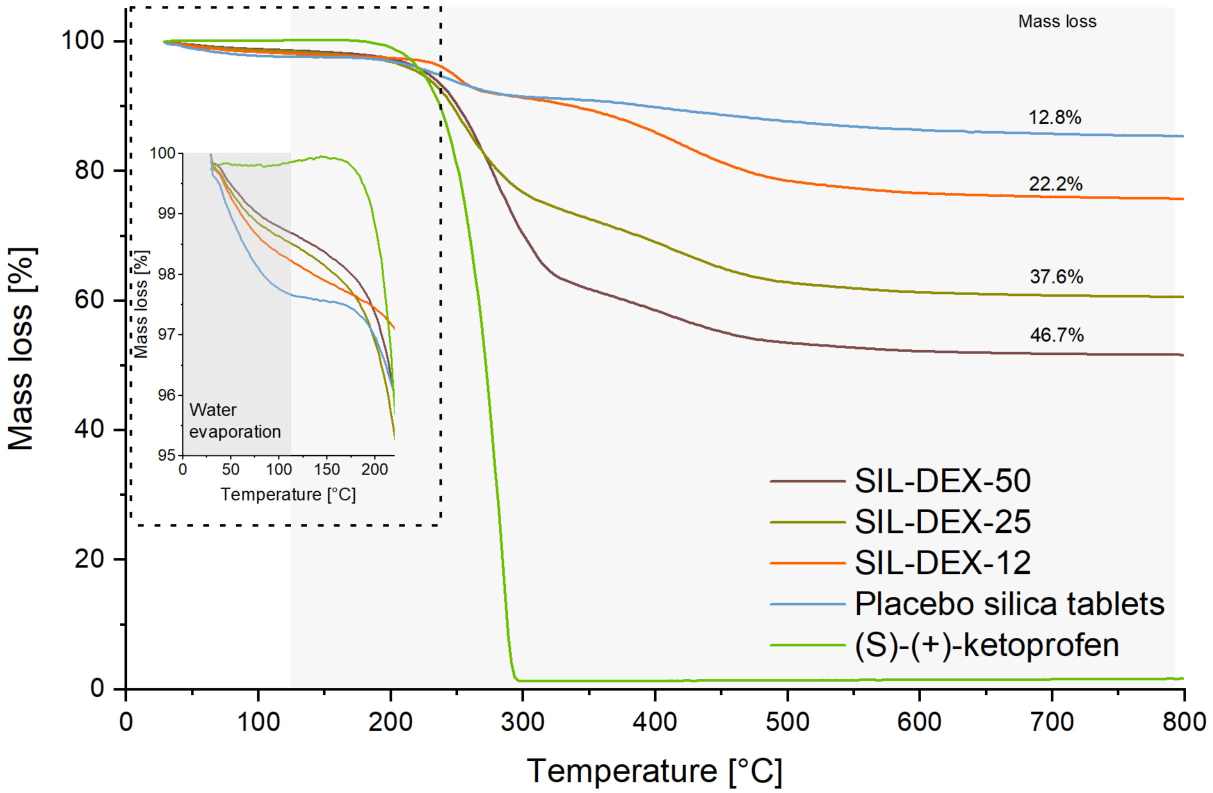

Figure S1. Thermogravimetric studies of (S)(+) ketoprofen, silica and silica tablets with (S)(+)ketoprofen

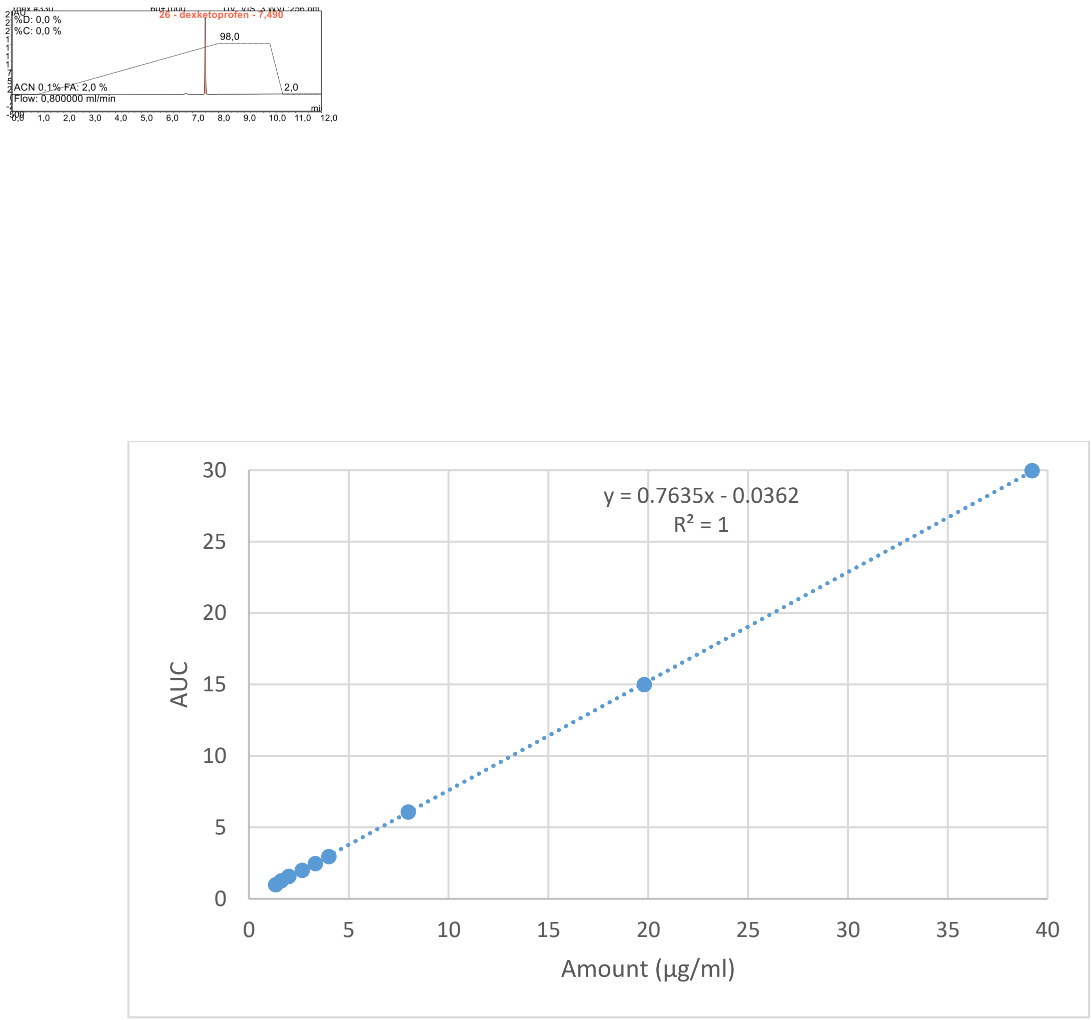

Figure S2. Top. HPLC chromatogram of the pure (S)(+) ketoprofen and bottom calibration curve used for HPLC determination of the released drug from silica monoliths.

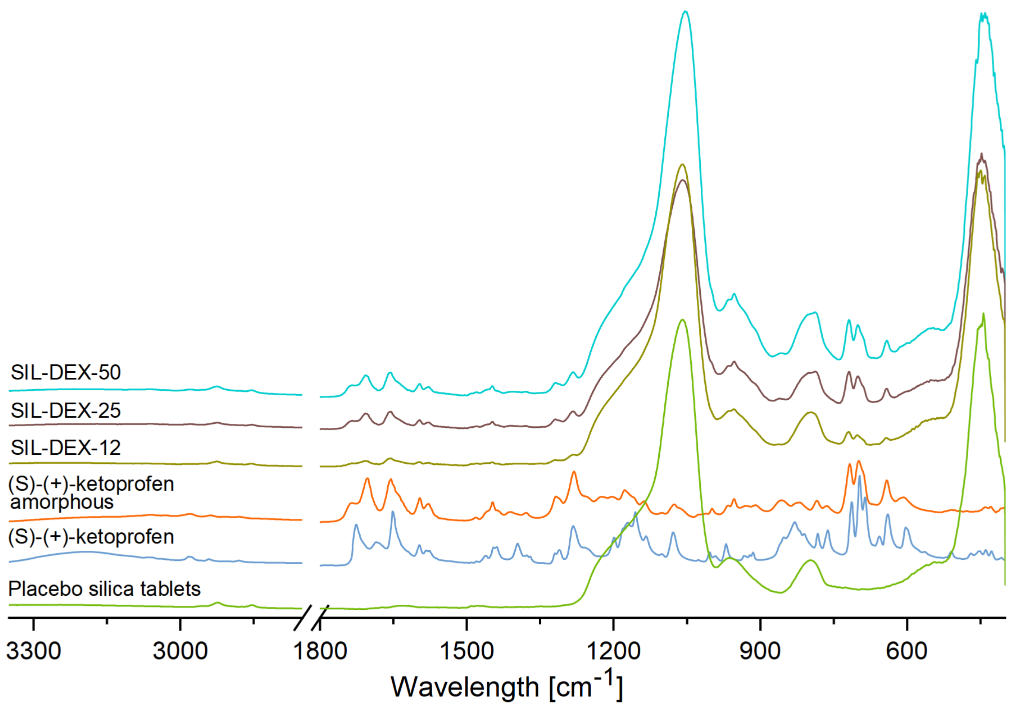

Figure S3. FTIR spectra of (s)-(+) ketoprofen, silica and silica tablets with three doses of (s)-(+)-ketoprofen

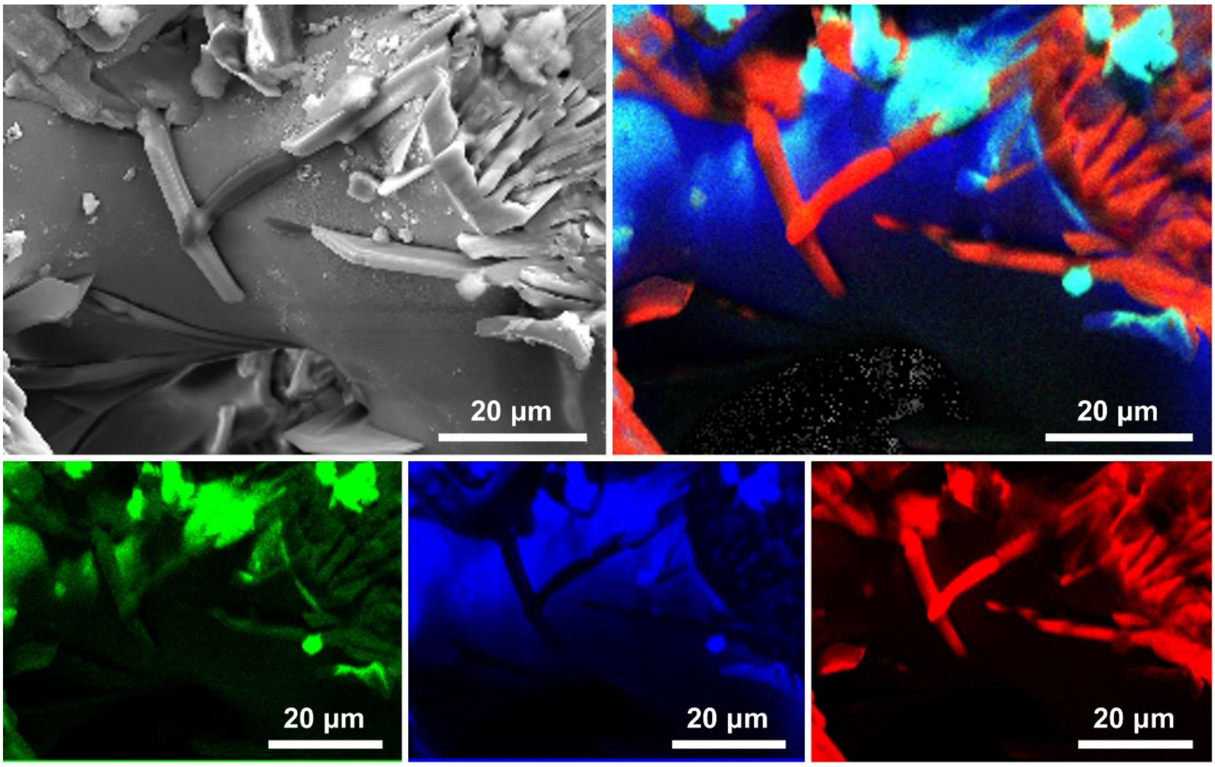

Figure S4. Mapping (SEM/EDX spectra) of macroporous silica skeleton with incorporated dexketoprofen. A – SEM micrograph of drug crystals incorporated on macropore's wall; B-E micrographs showing distribution of silicon (green), oxygen (blue) and carbon (red).

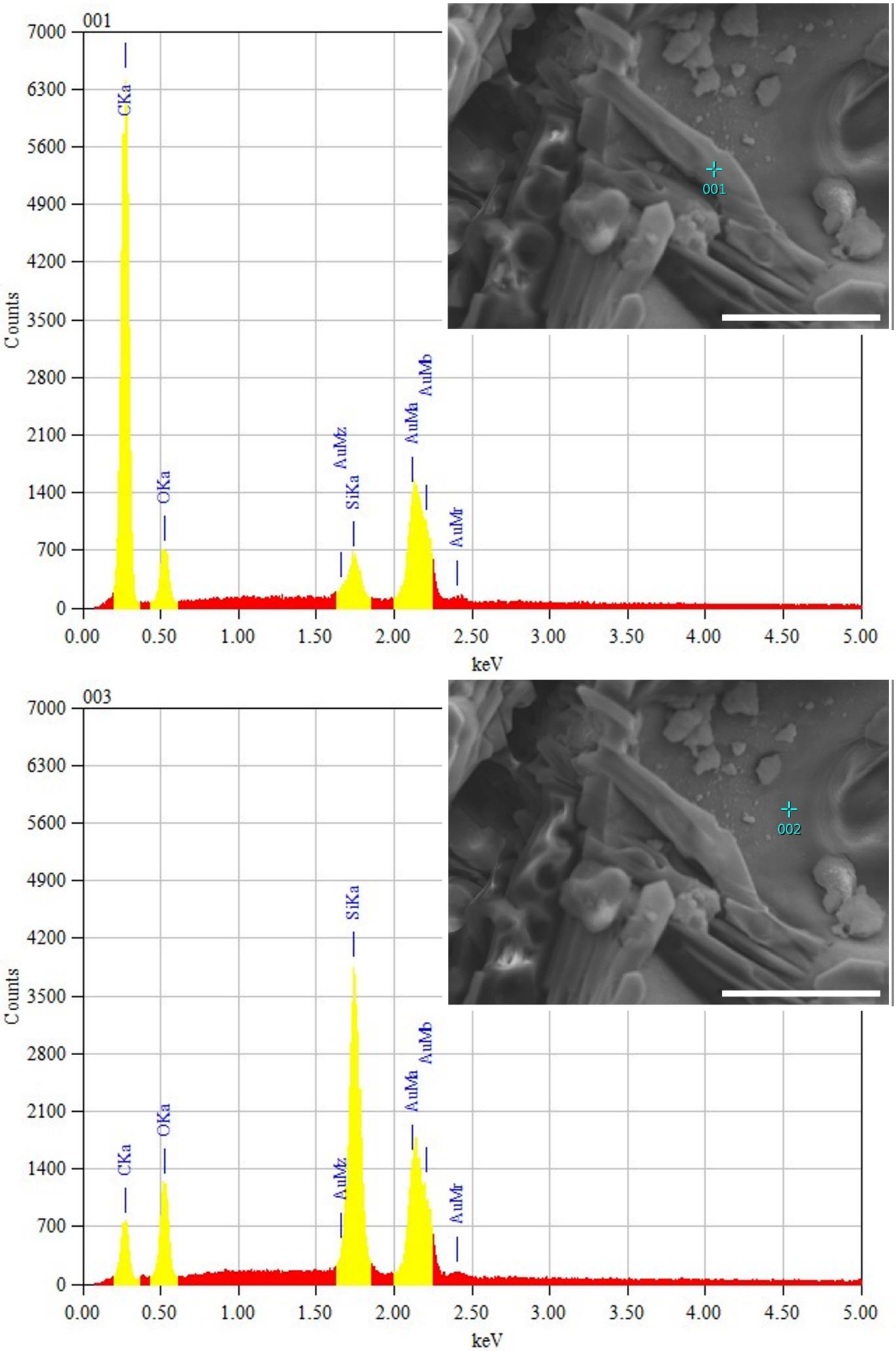

Figure S5. Focused distribution of carbon, oxygen and silicon in crystal (point 1) and outside of the crystal (on the wall) (point 2)

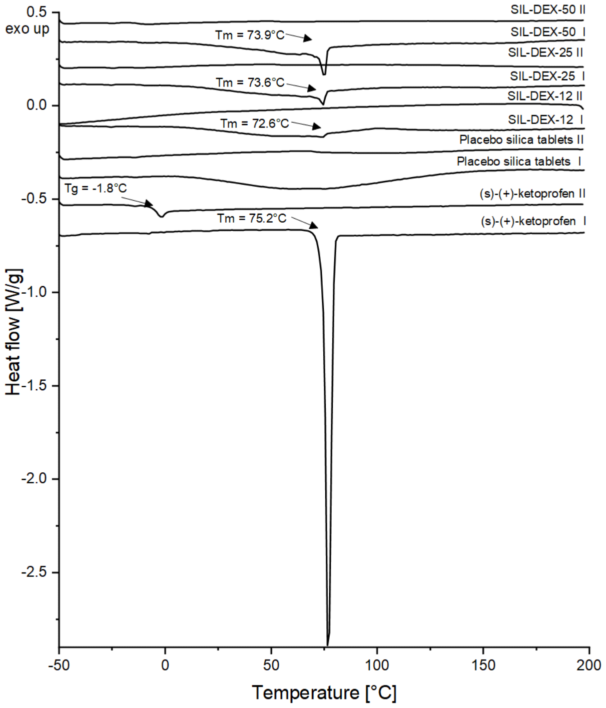

Figure S6. DSC thermogram of silica monolithic tablets with incorporated (s)-(+)-ketoprofen.

SECTION 2. Drug release modelling

The model assumes that the drug substance diffuses from a cylinder with finite dimensions: radius (*R*) and length (2 x *Z*) with a homogeneous initial concentration of the diffusing substance C_{in}, which is immersed in a strongly mixed acceptor fluid with an infinite volume maintaining the concentration of C_{ext}. In addition, it was assumed that the coefficient of transfer of matter to the acceptor fluid is so high that a constant concentration of the drug substance is maintained on the cylinder surface during the diffusion process, and equals to C_{∞}, where $C\infty = K \cdot C_{ext}$, and *K* is the partition coefficient matrix/acceptor fluid. Mathematically, the above initial and boundary conditions are defined:

$t = 0,$	$0 < r < R,$ $-Z < z < Z,$	$C = C_{in},$	cylinder of finite dimensions	(2)
$t > 0,$	$r = R,$ $z = \pm Z,$	C_{∞} = surface of a cylinder		(3)

$$C_{\infty} = K \cdot C_{ext}. \tag{4}$$

The solution used assumes that the diffusion process takes place in two planes: parallel to the radius of the cylinder and parallel to its length.

Finally, the solution of the diffusion equation (Equation 1) with the initial (Equation 2) and boundary (Equation 3) conditions defined in this way, which is the basis for the mathematical model used to evaluate the release of the drug substance from the tested matrices, takes the following form (Equation 5):

$$\frac{M_t}{M_\infty} = 1 - \frac{32}{\pi^2} \cdot \sum_{n=1}^{\infty} \frac{1}{q_n^2} \cdot exp\left[-\frac{q_n^2}{R^2} \cdot D \cdot t\right] \cdot \sum_{p=0}^{\infty} \frac{1}{(2 \cdot p+1)^2} \cdot exp\left[-\frac{(2 \cdot p+1)^2 \cdot \pi^2}{4 \cdot Z^2} \cdot D \cdot t\right], \tag{5}$$

where $\frac{M_t}{M_\infty}$ is the cumulative fraction of the released drug substance after time *t*, and q_n are successive zeros of the zero-order Bassel function: $J_0(q_n) = 0$.

Based on the obtained empirical data of drug substance release from the tested porous cylindrical matrices, the values of the diffusion coefficients *D* were determined by performing the least squares estimation based on the minimization of the loss function S (Equation 6) defined:

$$S = \sum(v.\ obs. - v.\ pred.)^2, \tag{6}$$

determined based on the Equation 5, where *v. obs* and *v. pred.* are observed and predicted values of the model, respectively.

The estimators of the diffusion coefficients *D* were calculated using the nonlinear Quqasi-Newton and Levenberg-Marquardt algorithms using the Mathematica® 12 software. In each of the three non-linear iterative procedures performed, the initial values for the diffusion coefficients were used as the D_0

estimators, determined using an approximate approximation of the equation 5 correctly describing the release process until the value of $\frac{M_t}{M_\infty} \leq 0{,}4$ (Equation 7):

$$\frac{M_t}{M_\infty} = 1 - \frac{32}{\pi^2} \cdot \left(\frac{1}{4} - \frac{1}{R} \cdot \sqrt{\frac{D_0 \cdot t}{\pi}}\right) \cdot \left(\frac{\pi^2}{8} \cdot \left(1 - \frac{2}{Z} \cdot \sqrt{\frac{D_0 \cdot t}{\pi}}\right)\right) \cdot . \qquad (7)$$

The initial step length was set at 0.001, the maximum number of iterations not exceeding 10,000, with a convergence criterion of $1 \cdot 10^{-9}$. Successive zeros of the zero-order Bassel function were determined using the FindRoot algorithm available in the Mathematica® software, assuming the following solutions to the equation as approximate initial values for these iterations (Equation 8):

$$q_n \approx \pi \cdot (n - 0.25), \text{ where } n \in N \qquad (8)$$

The calculations took into account the actual geometry of the porous monolith, which were in the shape of cylinders with average dimensions R = 0.1932 cm and H = 0.4932 cm (2 · *Z*). The results of the nonlinear estimation and the quality of the model fit (Equation 5) to the empirical data obtained are summarized in Table 2 and in the ESI Table S2.

Dissolution efficiency - D.E. defined as:

$$D.E. = \frac{AUC}{y_{100\%} \cdot t} = \frac{\int_0^t Q(t)dt}{100 \cdot t}, \qquad (9)$$

Where Q(t) is the percentage/fraction of the drug released from the formulation after time t.

In practice, the area under the AUC release profile was determined by the classical method of summing trapezoidal areas according to the formula:

$$\int_0^t Q(t)dt = \int_0^{t_n} Q_n(t)dt = \sum_{i=1}^{n} \frac{Q_{i+1}(t) + Q_i(t)}{2} \cdot (t_{i+1} - t_i). \qquad (10)$$

Finally:

$$D.E. = \frac{\sum_{i=1}^{n} \frac{Q_{i+1}(t) + Q_i(t)}{2} \cdot (t_{i+1} - t_i)}{100 \cdot t_{last}}. \qquad (11)$$

Table S2. Statistical evaluation of the nonlinear estimation performed using the least squares method based on the diffusion model (5). Part 2.

Formulation	*Observed values OV*	Predicted values PV	Standard error SE	Confidence interval for mean forecasts *PV - 95% CI – PV. + 95% CI*
**SIL-DEX-12**	0.29460	0.34134	0.01325	0.307278 - 0.375403
	0.45671	0.47128	0.01698	0.427635 - 0.514916
	0.64588	0.61575	0.01977	0.564935 - 0.666573
	0.75275	0.70812	0.02044	0.655568 - 0.760661
	0.85598	0.86148	0.01772	0.815927 - 0.907038
	0.90206	0.91405	0.01446	0.876867 - 0.951223
	0.91835	0.97912	0.00610	0.963450 - 0.994793
**SIL-DEX-25**	0.15929	0.21475	0.00578	0.201659 - 0.227831
	0.26030	0.30386	0.00787	0.286048 - 0.321662
	0.39610	0.41068	0.01006	0.387918 - 0.433445
	0.48871	0.48545	0.01135	0.459773 - 0.511125
	0.61062	0.63218	0.01308	0.602580 - 0.661771
	0.72890	0.69786	0.01338	0.667581 - 0.728131
	0.85868	0.82446	0.01249	0.796194 - 0.852720
	0.92260	0.89539	0.01038	0.871897 - 0.918878
	0.94751	0.93719	0.00804	0.919006 - 0.955381
	0.95421	0.96807	0.00533	0.956024 - 0.980119
	0.96006	0.98375	0.00335	0.976177 - 0.991313
**SIL-DEX-50**	0.08775	0.15485	0.00596	0.141573 - 0.168123
	0.15273	0.22137	0.00830	0.202884 - 0.239863
	0.24657	0.30340	0.01097	0.278960 - 0.327843
	0.31718	0.36263	0.01273	0.334265 - 0.390994
	0.42298	0.48480	0.01582	0.449540 - 0.520052
	0.53822	0.54306	0.01699	0.505213 - 0.580906
	0.68504	0.66642	0.01854	0.625115 - 0.707726
	0.78803	0.74847	0.01858	0.707057 - 0.789874
	0.85025	0.80761	0.01782	0.767891 - 0.847322
	0.90845	0.86403	0.01610	0.828163 - 0.899891
	0.93334	0.90337	0.01398	0.872220 - 0.934520
	0.94592	0.92507	0.01234	0.897587 - 0.952560

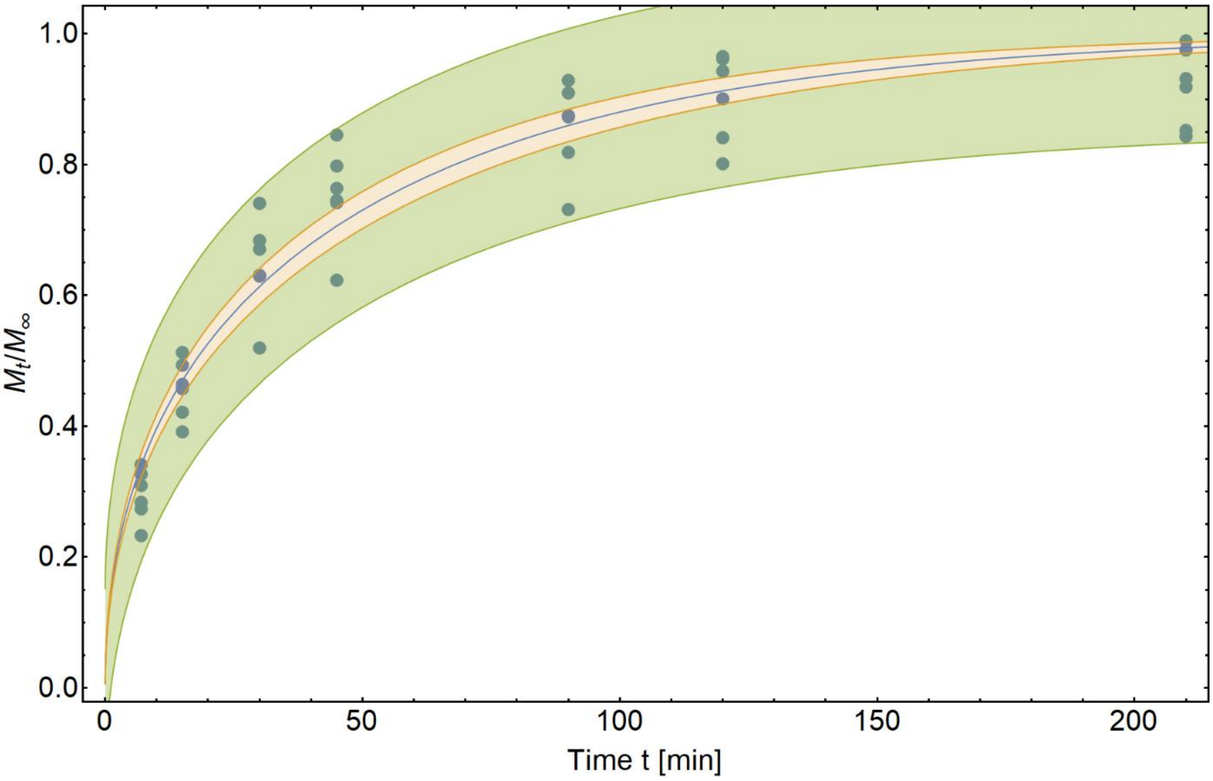

Figure S7. Fitting the diffusion model (5) to the raw drug substance release results from the SIL-DEX-12 matrix along with 95% confidence intervals for mean predictions and 95% confidence intervals for the predicted response from a single observation.

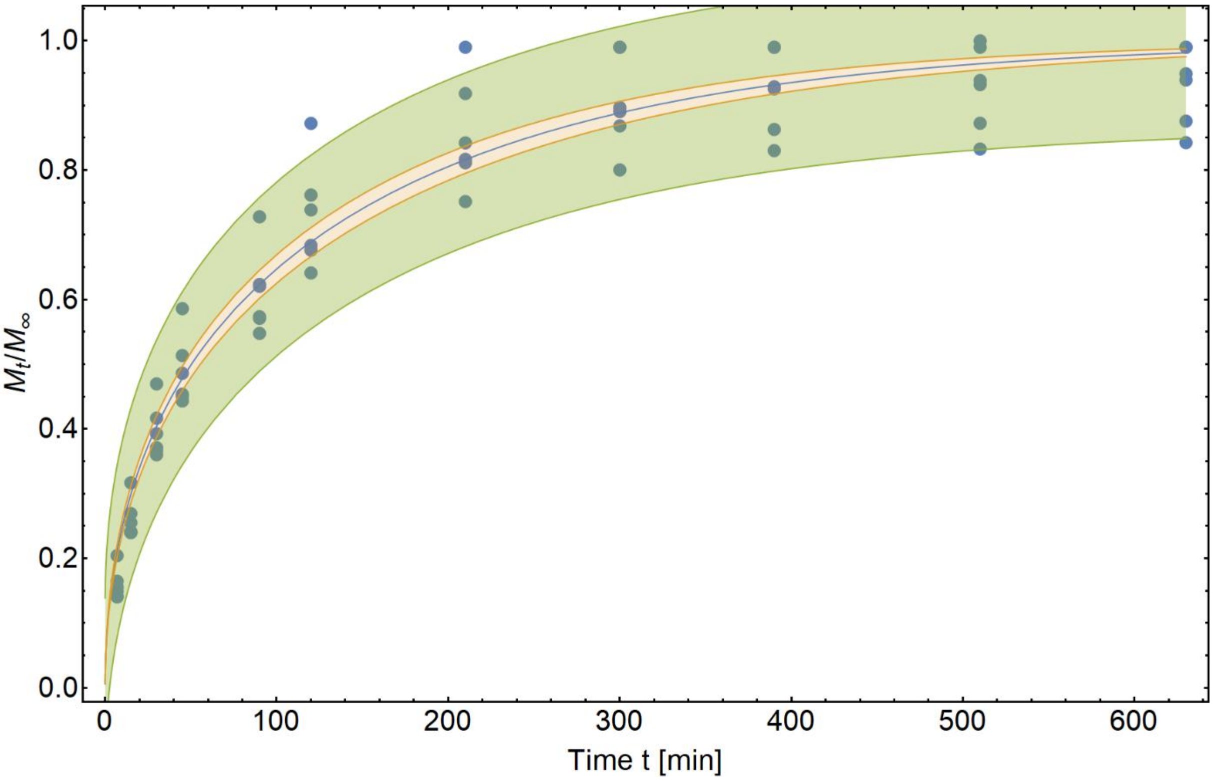

Figure S8. Fitting the diffusion model (5) to the raw drug substance release results from the SIL-DEX-25 matrix along with 95% confidence intervals for mean predictions and 95% confidence intervals for the predicted response from a single observation.

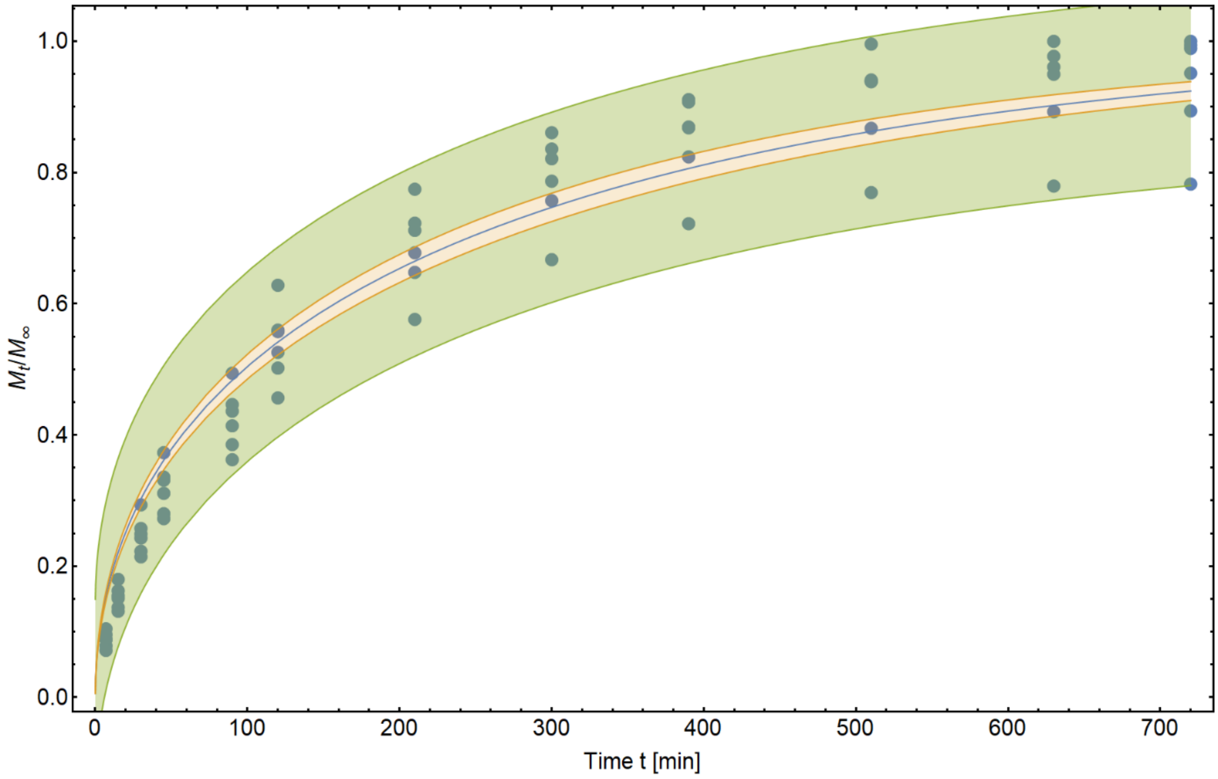

Figure S9. Fitting the diffusion model (5) to the raw drug substance release results from the SIL-DEX-50 matrix along with 95% confidence intervals for mean predictions and 95% confidence intervals for the predicted response from a single observation.

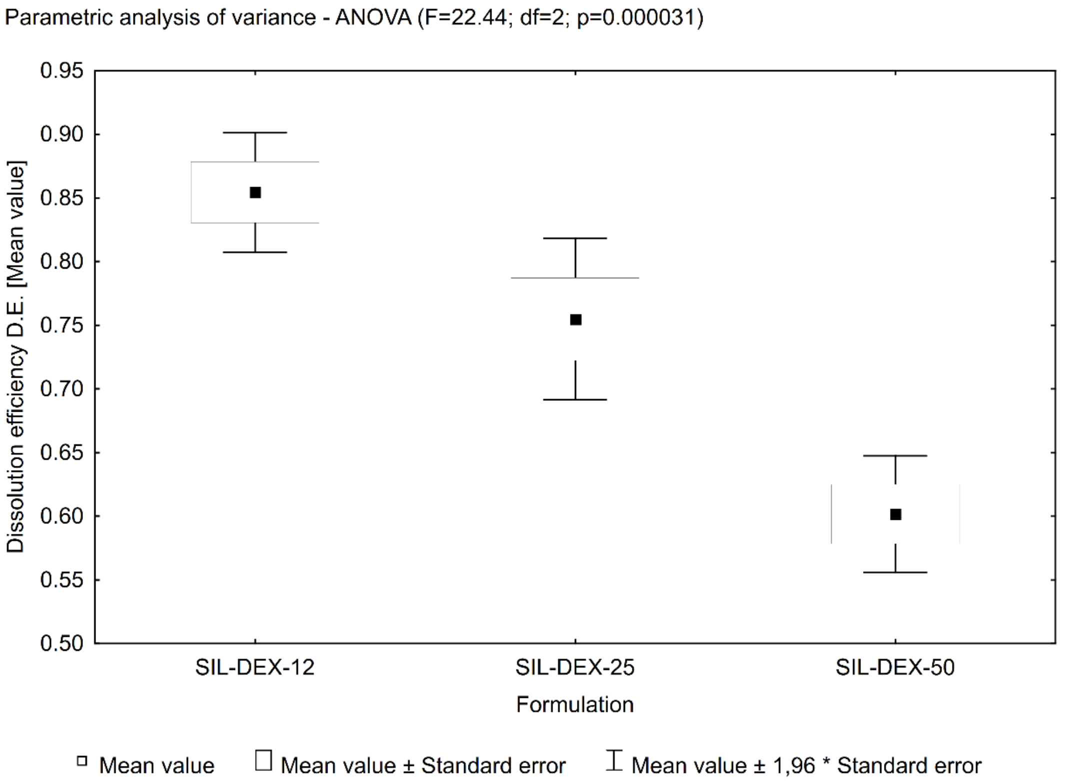

Figure S10. Parametric analysis of variance – ANOVA. Comparison of the mean values of the release efficiency determined for the three tested matrices – SIL-DEX-12; SIL-DEX-25; SIL-DEX-50.

Table S4. The results of post-hoc NIR Multiple Comparison Test (Fisher Least Significant Difference)

Formulation	Test NIR; The differences are significant with $p < 0.050$		
	**SIL-SEX-12**	**SIL-DEX-25**	**SIL-DEX-50**
**SIL-DEX-12**	-	0.019363 (+)	0.000008 (+)
**SIL-DEX-25**	0.019363 (+)	-	0.001089 (+)
**SIL-DEX-50**	0.000008 (+)	0.001089 (+)	-
